# Traumatic Cervical Spinal Cord Injury and Income and Employment Status

**DOI:** 10.1001/jamanetworkopen.2024.18468

**Published:** 2024-06-25

**Authors:** Rachael H. Jaffe, Peter C. Coyte, Brian C.-F. Chan, Rebecca L. Hancock-Howard, Armaan K. Malhotra, Karim Ladha, Jefferson R. Wilson, Christopher D. Witiw

**Affiliations:** 1 Institute for Health Policy, Management and Evaluation, University of Toronto, Ontario, Canada; 2 Division of Neurosurgery, St Michael’s Hospital, University of Toronto, Toronto, Ontario, Canada; 3 KITE-Toronto Rehabilitation Institute, University Health Network, University of Toronto, Toronto, Ontario, Canada; 4 Department of Anesthesiology and Pain Medicine, St Michael’s Hospital, University of Toronto, Toronto, Ontario, Canada

## Abstract

**Importance:**

Spinal cord injury (SCI) causes drastic changes to an individual’s physical health that may be associated with the ability to work.

**Objective:**

To estimate the association of SCI with individual earnings and employment status using national administrative health databases linked to income tax data.

**Design, Setting, and Participants:**

This was a retrospective, national, population-based cohort study of adults who were hospitalized with cervical SCI in Canada between January 2005 and December 2017. All acute care hospitalizations for SCI of adults ages 18 to 64 years were included. A comparison group was constructed by sampling from individuals in the injured cohort. Fiscal information from their preinjury years was used for comparison. The injured cohort was matched with the comparison group based on age, sex, marital status, province of residence, self-employment status, earnings, and employment status in the year prior to injury. Data were analyzed from August 2022 to January 2023.

**Main outcomes and Measures:**

The first outcome was the change in individual annual earnings up to 5 years after injury. The change in mean yearly earnings was assessed using a linear mixed-effects differences-in-differences regression. Income values are reported in 2022 Canadian dollars (CAD $1.00 = US $0.73). The second outcome was the change in employment status up to 5 years after injury. A multivariable probit regression model was used to compare proportions of individuals employed among those who had experienced SCI and the paired comparison group of participants.

**Results:**

A total of 1630 patients with SCI (mean [SD] age, 47 [13] years; 1304 male [80.0%]) were matched to patients in a preinjury comparison group (resampled from the same 1630 patients in the SCI group). The mean (SD) of preinjury wage earnings was CAD $46 000 ($48 252). The annual decline in individual earnings was CAD $20 275 (95% CI, −$24 455 to −$16 095) in the first year after injury and CAD $20 348 (95% CI, −$24 710 to −$15 985) in the fifth year after injury. At 5 years after injury, 52% of individuals who had an injury were working compared with 79% individuals in the preinjury comparison group. SCI survivors had a decrease in employment of 17.1 percentage points (95% CI, 14.5 to 19.7 percentage points) in the first year after injury and 17.8 percentage points (14.5 to 21.1 percentage points) in the fifth year after injury.

**Conclusions and Relevance:**

In this study, SCI was associated with a decline in earnings and employment up to 5 years after injury for adults aged 18 to 64 years in Canada.

## Key Points

**Question:**

Is a spinal cord injury associated with changes in an individual’s income and employment status?

**Findings:**

In this cohort study of 1630 adults with cervical spinal cord injury, there was a statistically significant decline in earnings and employment status in the 5 years after injury.

**Meaning:**

In this study, spinal cord injury survivors sustained a large loss in income and employment after injury.

## Teaser

This cohort study investigates the association of spinal cord injury with individual earnings and employment status up to 5 years after injury among patients in Canada.

## Introduction

Traumatic spinal cord injury (SCI) is a life-altering event associated with tremendous personal loss, direct health care resource requirements, and challenges with societal reintegration.^1^ SCI incidence peaks in young adulthood, when individuals are in their prime wage-earning years.^2,3,4^ The labor market implications of traumatic SCI are poorly understood but represent an essential dimension of postinjury stressors for patients. Increased absenteeism and loss in productivity are consequences of decreased mental and physical function after an SCI.^5,6,7,8^

Furthermore, potential economic consequences may be substantial, such as reduced governmental income tax revenue and increased pressure on social services, including unemployment insurance or disability payments.^9,10^ At an individual level, unemployment is associated with depression, anxiety, and a decrease in self-esteem.^11,12^ Studies have demonstrated that financial insecurity may hinder the recovery of a patient with an SCI through increased individual stress, decreased access to health services, and caregiver burnout.^13,14^ In contrast, employment is important for financial security, increases independence, strengthens social relationships, and enhances a sense of self-worth.^1,15,16,17^

Understanding outcomes associated with SCI and how to minimize income and employment losses after injury is important. Previous research showed that the decline in income and employment after injury was significant. An Australian study^18^ showed that close to 50% of survey respondents who had an SCI were unable to perform paid work after injury. A study in the Netherlands, Denmark, Norway, and Switzerland^19^ also showed that approximately half of survey respondents were not employed after an SCI. A cross-sectional study set in the southeastern US^20^ also found a decrease in employment after SCI and that injury severity was associated with greater income loss. Because of the nature of survey data, results of these studies can be highly biased due to small sample sizes and self-reported data. These studies also failed to assess longitudinal outcomes and often lacked adjustment for confounding.^18,19,20,21^

These limitations in existing literature underscore the importance of quantifying postinjury employment and income loss among patients experiencing traumatic SCI. Consequently, the purpose of this study was to assess the association of traumatic SCI with earnings and employment using a national, population-based observational cohort. We hypothesized that there would be a substantially negative association of SCI with work and earnings in the short and long term.

## Methods

### Study Design

We performed a retrospective, population-based cohort study that used a difference-in-difference (DID) study design to obtain estimates of the association of acute traumatic cervical SCI with individual employment income and employment status among injury survivors. This research was approved by the Research Ethics Board at Unity Health Toronto, Toronto, Ontario, Canada. All data were deidentified, and therefore Unity Health Toronto determined that informed consent was not required for data access and analysis. This study followed the Strengthening the Reporting of Observational Studies in Epidemiology (STROBE) reporting guideline.

### Datasets and Linkage

The public health care system in Canada funds all medically necessary health care services for Canadian citizens. This allows for the compilation of administrative databases that include the health care use of most of the population. Income, employment, and health data of all patients with SCI in Canada were derived from linkage between the Canadian Institute for Health Information Discharge Abstract Database (CIHI-DAD) and the T1 Family File (T1FF), completed using the Derived Record Depository in the Social Data Linkage Environment of Statistics Canada.^22^ The CIHI-DAD captures all acute care hospitalizations in the country, except for those in the province of Quebec, for the fiscal years of 2004 to 2005 through 2019 to 2020.^23^ Longitudinal individual tax information was derived from the T1FF provided by the Canada Revenue Agency.^24^ The linked database includes all individuals who have filed taxes in Canada and have been hospitalized in any province in Canada other than Quebec between 2004 and 2019 (eMethods 1 in Supplement 1).

### Study Population

The study population was composed of individuals aged 18 to 64 years who were hospitalized in Canada for an acute traumatic cervical SCI between January 1, 2005, and December 3, 2017. The younger age cutoff used limits this study to adults, while the upper limit was selected to avoid typical retirement age effects. Additionally, in the age 18 to 64 years group included in this study, approximately 88% of individuals file taxes with individual earnings predominantly (80%) derived from employment.^25,26^ Given that the CIHI-DAD does not capture any individuals who were hospitalized in Quebec, our study cohort excludes individuals who were treated in Quebec.

Cases were identified by *International Statistical Classification of Diseases and Related Health Problems, Tenth Revision-Canada* (*ICD-10-CA*) diagnostic codes for cervical SCI; 4270 patients were identified (eTable 1 in Supplement 1).^27^ Patients who were hospitalized with injuries from burns, poisonings, drownings, exposure, suffocation, overexertion, submersion, or unspecified mechanisms were excluded because they do not usually use regional trauma system resources and are commonly excluded in studies evaluating injuries related to trauma.^28,29^ Additionally, we excluded 510 individuals who were admitted with any prior traumatic injuries, spinal cord related or not, in the 2 years prior to the index year to ensure that we excluded individuals potentially readmitted due to complications associated with prior injuries (11.9%). We also excluded 290 individuals who died before discharge (6.7%) and 40 individuals with missing income or demographic information (1.2%) (eFigure 1 in Supplement 1).

### Comparison Group

We identified candidate comparator patients from our main cohort; these individuals were sampled in their preinjury years to reduce the potential for unmeasured confounding.^30^ Given that all members of the comparison group would eventually sustain a traumatic SCI, we assumed that they had similar preinjury characteristics to the exposed cohort. This served to reduce confounding of the outcome by ensuring that unmeasured characteristics, such as an individual’s relationship with risk, were captured. With this method, we could support the parallel trend assumption of a DID design by creating a valid counterfactual for what we would have observed if those individuals had never experienced an SCI.

We anchored comparator individuals using the index year of injury from the main cohort, such that comparator injuries occurred 6 years after the index year. For example, we compared data for individuals with an injury in 2010 with the 2010 data of those who had an injury in 2016 or later. For individuals in the comparison group, their preinjury data were recoded to have a matching index year (Y 0) as the year of injury of individuals who were injured (eFigure 2 in Supplement 1). Individuals who sustained injuries in 2013 or later could be compared only with individuals who sustained a traumatic SCI injury in 2019. We chose a study period of 7 years, from Y −2 to Y 5, because the 2 years prior to injury helped to establish a preinjury trend for analysis and prior studies suggest that SCI survivors are able to partially regain employment by the fifth year after injury.^31,32,33^ Finally, we matched patients in the injured group with those in the comparison group on baseline (Y −1) demographics (age, sex, and province of tax filing) and baseline earnings–associated variables (earnings in Y −1, employment status in Y −1, and self-employment status). Matching was performed using coarsened exact matching (eMethods 2 in Supplement 1).^34,35^

### Outcomes

We defined employment as having any nonzero earnings based on the employment income recorded in tax filings from the T1FF file, excluding income from assets and investments. Total employment income includes wages, salaries, and commissions from employment; training allowances; tips; and gratuities. It also includes any income from self-employment.^36^ Individuals who file taxes with negative income (indicating net losses from self-employment) were included in the analysis given that they have positive employment status; however, their income was set to zero to reduce downward bias in final model estimates for income.^30,37^ All income values are reported in 2022 Canadian dollars (CAD $1.00 = US $0.73).^38^ All income values were rounded to the nearest CAD $1000 due to data source restrictions.

We studied 2 outcomes of interest. First, we assessed the change in income for each year over the 5-year period after injury. Second, we assessed the change in the proportion of employed individuals for each year of the postinjury period.

### Statistical Analysis

To evaluate the parallel-trends assumption for individual income and employment status, we ran an event study estimation model to assess the difference in exposed and comparison group outcomes at times prior to injury.^39,40^ We found that there were no significant differences between preinjury trends in comparison and exposed groups for earnings or employment status (eFigure 3 in Supplement 1).

The association of SCI with income was estimated with a mixed-effects multivariable regression model, adjusting for individual-level fixed effects over time. We quantified the change in mean yearly earnings as a function of exposure group status, preinjury or postinjury period, and interaction between time and injury status. The interaction term is the DID estimate. There were 2 exogenous outcomes that were of concern in the analysis. First, individuals typically experience rapid wage growth in their mid-20s to early 30s, which then stabilizes in their later years (ages 40-50 years).^30^ Second, broad economic factors create variability in annual earning; thus, it is possible that an economic cycle may be associated with wages and thereby bias the association of a traumatic SCI with income and employment. We adjusted for the current age of the individual as a continuous variable and included the fiscal year as a categorical variable in all models (eMethods 3 in Supplement 1).

All estimates of changes in earnings are reported as absolute differences from the year prior to injury (Y −1) to 1 to 5 years after injury. Absolute differences in income are mean annual changes in income reported as a dollar value (CAD $).

The association of SCI with employment status was estimated with a generalized mixed-effects multivariable probit regression model, with a DID estimator. For this model, we also controlled for the fiscal year and age during the fiscal year. We report estimates for the change in employment status as the mean marginal effect or the difference in percentage employed between the injured and comparison group when all other covariates are at their population mean, reported as percentage points.

We performed a series of prespecified secondary analyses. First, to assess the association of early retirement with earnings and employment status after SCI, we restricted the overall cohort to individuals aged 53 years or younger in their index year.^28^ Age 53 years was a conservative cutoff to capture anyone retiring prior to the mean age of retirement in Canada, which was approximately 60 years between 2005 and 2019.^41^ Second, to examine the association of prior employment with income, we looked at the association of employment with income in the years leading up to an individual’s injury by restricting the analysis to individuals who were employed in Y −1 and Y −2. Additionally, we wanted to explore the association of more severe injuries with labor market outcomes. We stratified the cohort into complete and incomplete SCI by *ICD-10-CA* code (eTable 1 in Supplement 1). All analyses were completed using R statistical software version 4.1.2 (R Project for Statistical Computing).^42^ We set an a priori level of significance of a 2-sided *P* < .05. Data were analyzed from August 2022 to January 2023.

## Results

### Sample Characteristics

We identified 1630 patients (mean [SD] age, 47 [13] years; 1304 male [80.0%]) hospitalized with cervical SCI between 2005 and 2017 who were not previously hospitalized for any traumatic injury in the 2 years prior to the index year (Table 1; eTable 2 in Supplement 1). Most injuries were attributable to falls and motor vehicle traffic accidents (Table 1). The comparison group was resampled from the same 1630 patients in the SCI group.

**Table 1.** Individual and Injury Characteristics

Characteristic^a^	Patients, No. (%) (N = 1630)
Individual	
Age, mean (SD), y	47 (13)
Urban residency	1255 (76.99)
Sex	
Male	1304 (80.00)
Female	326 (20.00)
Income, mean (SD), CAD $^b^	
Y −2	47 000 (73 866)
Y −1	48 000 (91 817)
Family size, mean (SD), No. individuals	2.50 (1.41)
Married	783 (48.03)
Self employed	196 (12.02)
Injury	
Discharge disposition	
Transferred to long-term care facility	650 (39.87)
Discharged to home setting	603 (36.99)
Transferred to another facility providing inpatient care	293 (17.97)
Discharged to home setting with support services	49 (3.01)
Transferred to other or signed out (against medical advice)	33 (2.02)
Province of injury	
Ontario	668 (40.98)
British Columbia	424 (26.01)
Alberta	212 (13.01)
PEI, NL, NS, or NB	114 (6.99)
Manitoba	98 (6.01)
Length of stay, mean (SD), d	27 (74.55)
Responsibility for payment	
Province or territory with responsibility^c^	1500 (92.02)
WCB or WSIB, other province or territory (resident of Canada), Canadian resident self-pay, or other^c^	130 (7.98)
Mechanism of injury	
Fall	848 (52.02)
Motor vehicle traffic	293 (17.97)
Struck by object, cut, or pierced	163 (10.00)
Other transportation	114 (6.99)
Pedal cyclist (including pedestrian)	98 (6.01)
Natural or environmental, machinery, firearm, or other	130 (7.98)
Complete injury^d^	212 (13.00)

Abbreviations: NB, New Brunswick; NL, Newfoundland; NS, Nova Scotia; PEI, Prince Edward Island; WCB, Workers’ Compensation Board; WSIB, Workplace Safety and Insurance Board; Y, year.

^a^ All variables were assessed at the corresponding exposure year or the index year if among the exposed group.

^b^ Income values are reported in 2022 CAD (CAD $1.00 = US $0.73).

^c^ The territories consist of the Northwest Territories, Yukon, and Nunavut.

^d^ Complete injury refers to an American Spinal Injury Association Impairment Scale grade A injury.

### Change in Earnings After SCI

**Figure 1.**
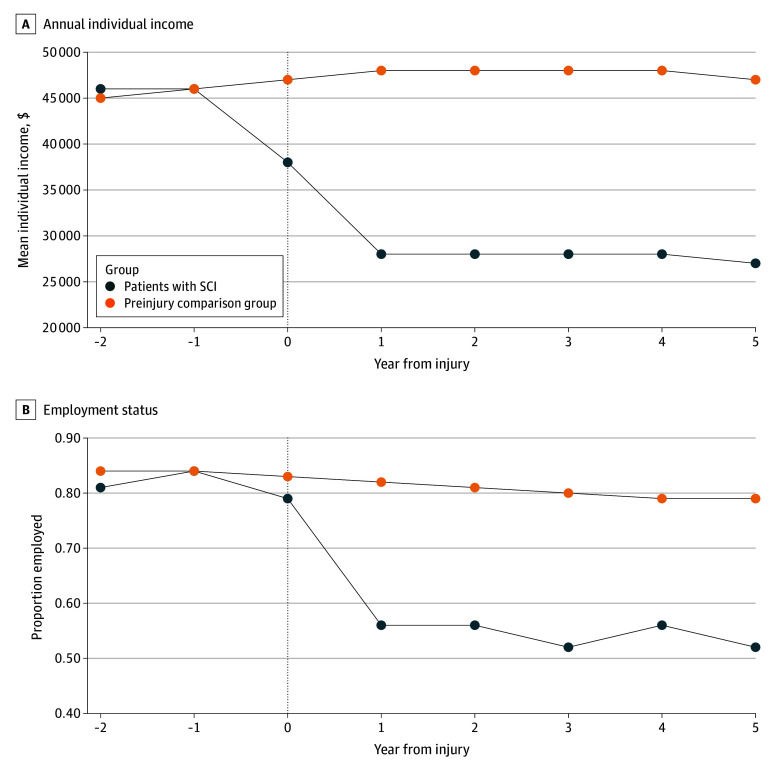
Income and Employment Over Time for Patients Matched in Year 1 The weighted mean annual income and proportion of employed individuals for patients with spinal cord injury (SCI) and the preinjury comparison group are shown.

In the year prior to injury, individuals with an SCI were earning a mean (SD) of CAD $46 000 ($49 262) annually and experienced a reduction in earnings in the 5 years after injury to a mean (SD) of CAD $27 000 ($43 055), a 41.3% reduction (Table 2; eTable 3 and eFigure 4 in Supplement 1). The mean reduction in annual individual earnings associated with injury was CAD $20 275 (95% CI, −$24 455 to −$16 095) in the first year after injury and CAD $20 348 (95%CI, −$24 710 to −$15 985) in the fifth year after injury (Table 2; Figure 1).

**Table 2.** DID in Individual Earnings and Employment for Patients Matched in Year 1

Year from injury	Patients with SCI (N = 1630)	Comparison group (n = 1630)	DID (95% CI)^a^
**Weighted annual income, mean (SD), $ CAD** ^b^			
−1	46 000 (49 262)	46 000 (43 555)	NA
1	28 000 (47 357)	48 000 (60 886)	−20 275 (−24 455 to −16 095)
2	28 000 (44 353)	48 000 (61 644)	−20073 (−24 467 to −15 679)
3	28 000 (42 586)	48 000 (62 445)	−20 561 (−25 412 to −15 709)
4	28 000 (43 271)	48 000 (63 772)	−19 727 (−24 980 to −14 472)
5	27 000 (43 055)	47 000 (50 377)	−20 348 (−24 710 to −15 985)
**Employed individuals, %**			
−1	84	84	NA
1	56	82	−17.1 (−19.7 to −14.5)
2	56	81	−17.5 (−20.2 to −14.8)
3	52	80	−19.5 (−22.4 to −16.6)
4	56	79	−16.9 (−20.1 to −13.8)
5	52	79	−17.8 (−21.1 to −14.5)

Abbreviations: DID, difference in difference; NA, not applicable.

^a^ DID from the year prior to injury to the year indicated for patients with injuries and comparison participants was derived from multivariable modeling.

^b^ Income values are reported in 2022 CAD (CAD $1.00 = US $0.73).

Our findings were robust to a cohort restricted to individuals aged 53 years and younger at the time of injury. In the fifth year after injury, these individuals had a mean reduction in annual earnings associated with injury of CAD $23 331 (95% CI, −$29 923 to −$16 738) (Table 3). The change in earnings was larger when the cohort was restricted to individuals who worked in the 2 years prior to injury. In this case, the mean change in earnings in the fifth year after injury was CAD $33 414 (95% CI, −$39 977 to −$26 851) (Table 3).

**Table 3.** DID in Individual Earnings and Employment for Subgroup Analyses

Year from injury	Patients with SCI	Comparison group	DID (95% CI)^a^
**Weighted annual income, mean (SD), $ CAD** ^b^			
Age ≤53 y			
−1	47 000 (71 942)	47 000 (87 449)	NA
1	32 000 (59 161)	51 000 (109 980)	−21 516 (−27 068 to −15 964)
2	33 000 (56 467)	53 000 (116 125)	−21 438 (−27 180 to −15 696)
3	33 000 (60 178)	53 000 (112 925)	−23 091 (−29 586 to −16 595)
4	35 000 (61 217)	54 000 (113 492)	−22 690 (−29 900 to −15 479)
5	35 000 (63 362)	54 000 (104 794)	−23 331 (−29 923 to −16 738)
Employed in Y −2 and Y −1			
−1	64 000 (103 001)	70 000 (98 671)	NA
1	40 000 (89 293)	73 000 (108 144)	−28 439 (−32 985 to −23 893)
2	39 000 (95 961)	74 000 (118 983)	−29 285 (−33 876 to −24 694)
3	39 000 (91 713)	73 000 (117 168)	−31 107 (−36 926 to −25 288)
4	38 000 (79 401)	72 000 (110 459)	−31 552 (−37 737 to −25 368)
5	37 000 (75 136)	73 000 (117 069)	−33 414 (−39 977 to −26 851)
Incomplete SCI			
−1	43 300 (40 302)	42 300 (37 305)	NA
1	27 500 (38 552)	45 600 (43 656)	−19 216 (−22 698 to −15 732)
2	28 600 (41 464)	45 900 (85 314)	−18 701 (−24 167 to 13 235)
3	29 800 (40 874)	45 400 (46 308)	−18 029 (−21 960 to −14 098)
4	30 300 (42 618)	46 700 (44 992)	−17 922 (−22 139 to −13 704)
5	28 800 (42 389)	47 000 (47 103)	−19 512 (−24 281 to −14 744)
Complete SCI			
−1	38 600 (35 999)	42 400 (36 132)	NA
1	9800 (24 664)	39 100 (37 505)	−28 142 (−35 583 to −20 700)
2	11 000 (27 636)	41 600 (39 362)	−28 404 (−36 858 to −19 950)
3	9000 (23 123)	46 400 (42 975)	−34 414 (−44 659 to −24 289)
4	11 100 (27 972)	42 000 (42 614)	−28 551 (−38 242 to −18 860)
5	12 900 (30 046)	45 100 (41 997)	−29 368 (−38 989 to −19 745)
**Employed individuals, %**			
Age ≤53 y			
−1	81	82	NA
1	55	81	−19.6 (−22.2 to −16.9)
2	55	80	−19.2 (−22.0 to −16.4)
3	54	80	−20.2 (−23.1 to −17.3)
4	56	80	−18.6 (−21.7 to −15.5)
5	56	80	−18.2 (−21.4 to −14.9)
Employed in Y −2 and Y −1			
−1	100	100	NA
1	67	99	−12.1 (−12.7 to −11.0)
2	64	98	−12.5 (−13.0 to −11.3)
3	62	98	−12.1 (−12.6 to −10.9)
4	61	97	−11.6 (−12.1 to −10.4)
5	60	97	−10.9 (−11.4 to −9.8)
Incomplete SCI			
−1	84	84	NA
1	59	81	−15.3 (−18.4 to −12.3)
2	59	80	−16.3 (−19.6 to −13.0)
3	55	79	−18.5 (−22.0 to −14.9)
4	59	81	−16.2 (−19.9 to −12.4)
5	57	81	−17.9 (−21.8 to −14.1)
Complete SCI			
−1	92	92	NA
1	31	82	−29.8 (−35.9 to −23.6)
2	27	77	−27.7 (−34.1 to −21.3)
3	25	80	−29.4 (−35.9 to −22.7)
4	33	77	−24.7 (−31.6 to −17.6)
5	34	78	−23.9 (−30.8 to −16.9)

Abbreviations: DID, difference in difference; NA, not applicable; SCI, spinal cord injury; Y, year.

^a^ DID estimates from the year prior to injury to the year indicated for patients with injuries and participants in the comparison group were derived from multivariable modeling.

^b^ Income values are reported in 2022 CAD (CAD $1.00 = US $0.73).

**Figure 2.**
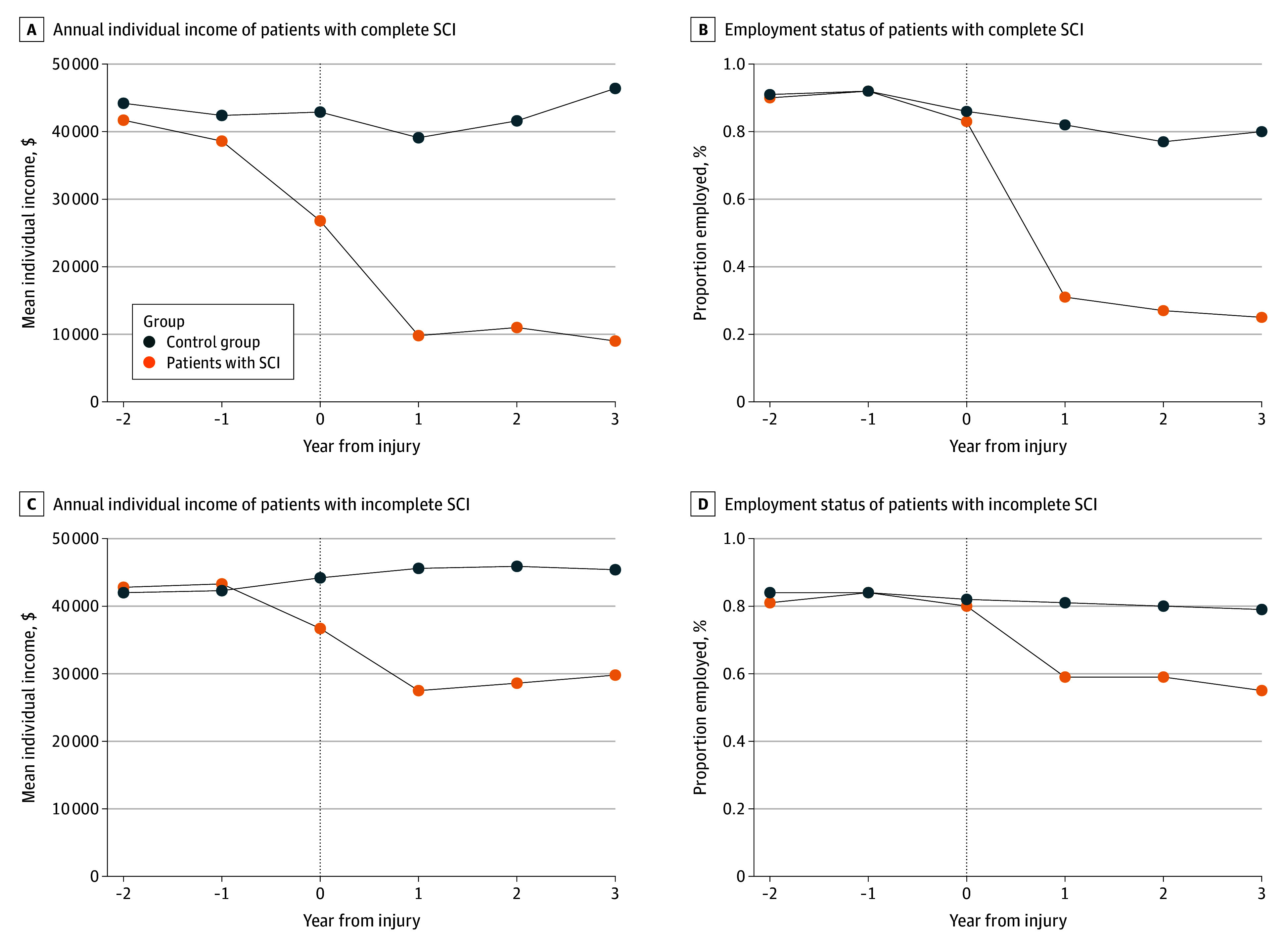
Income and Employment Over Time for Patients With Complete vs Incomplete Injury The weighted mean annual income and proportion of employed individuals over time for patients with incomplete and complete spinal cord injury (SCI) are shown.

When we stratified by injury severity, our results showed that individuals with a complete injury lost more income than those with an incomplete injury. In the fifth year after injury, individuals with a complete SCI had a mean reduction of CAD $29 368 (95% CI, −$38 989 to −$19 745), while those with an incomplete SCI had a mean reduction of CAD $19 512 (95% CI, −$24 281 to −$14 744) (Table 3; Figure 2).

### Employment Status After SCI

The proportion of individuals with injuries who were employed decreased from 84% in the year prior to injury to 52% in the fifth year after injury (Table 2). Meanwhile, individuals in the comparison group experienced a 5–percentage point decrease in employment (84% to 79%) (Table 2; eTable 3 and eFigure 4 in Supplement 1). Among patients with an SCI, employment decreased 17.1 percentage points (95% CI, 15 to 20 percentage points) in the first year after injury and 17.8 percentage points (15 to 21 percentage points) in the fifth year after injury. We estimated a significant reduction in the proportion of employed individuals 5 years after injury compared with the preinjury control group (17.8 percentage points; 95% CI, −21.1 to −14.5 percentage points) (Table 2; Figure 1).

Results were robust to secondary analyses; there were significant reductions in the proportion of employed individuals when the cohort was limited to those who were aged 53 years and younger at the time of injury and to individuals working throughout the 2 years prior to injury (Table 3). For individuals aged 53 years and younger, the mean decrease in the proportion employed in the fifth year after injury was 18.2 percentage points (95% CI, −21.4 to −14.9 percentage points). For individuals working throughout the 2 years prior to injury, the mean decrease in the proportion employed in the fifth year after injury was 10.9 percentage points (95% CI, −11.4 to −9.8 percentage points) (Table 3).

For individuals with a complete SCI, there were significant reductions in the proportion employed compared with individuals with an incomplete SCI. In the fifth year after injury, 34% of individuals with a complete SCI were employed, while 57% of individuals with an incomplete SCI were employed (Table 3). The mean decrease in the proportion employed in the fifth year after injury was 23.9 percentage points (95% CI, −30.8 to −16.9 percentage points) for patients with a complete SCI compared with 17.9 percentage points (95% CI, −21.8 to −14.1 percentage points) for patients with an incomplete SCI (Table 3; Figure 2).

## Discussion

This national longitudinal cohort study using linked individual health care and income tax data found that traumatic cervical SCI was associated with substantial societal outcomes, including changes in income and employment over a 5-year postinjury time horizon. Individuals who were injured had a 41.3% decline in mean income from the year prior to injury in the 5 years after injury. We also found a significant change in employment after injury compared with individuals without an injury. The proportion of employed individuals in the 5 years after injury was 32 percentage points lower than that before injury. These findings were robust to a subgroup analysis of individuals in their prime wage-earning years and of individuals with complete tax return information in the 2 preinjury years.

Our findings showed that individuals with a complete SCI had greater income loss and were less likely to be employed after injury. Many studies have shown that severity of injury was associated with lower rates of employment after injury.^18,33,43,44^ Due to lower levels of physical and mental functionality, an individual with tetraplegia from a complete SCI may experience more physical challenges and complications, such as spasticity, cardiorespiratory dysfunctions, and poorer hand functions, which can inhibit their ability to return to work.^44,45^

Our results demonstrated that prior employment had a protective association against the magnitude of postinjury loss of employment. Previous studies have seen similar results, indicating that individuals with SCI who were employed prior to injury may return faster to the labor market because they may be able to return to the original place of work or are predisposed to other employment opportunities.^18,19,28,46,47^ Among other injury and individual characteristics, such as education level, we might expect that the change in labor market outcomes after injury are highly dependent on prior employment and preinjury income.^28^

Most prior studies that explored the association of SCI with labor market outcomes have been survey based.^18,19,20,21^ These studies were completed in the US,^20^ Australia,^18^ and Europe^19,21^ and found that approximately 40% to 50% of SCI survivors were able to return to work after injury. A 2023 population-based study from Norway by Halvorsen et al^31^ used an SCI registry to estimate labor market participation in the 6 years after injury. That study found that in the year of injury, 100% of the SCI cohort was employed, which decreased to approximately 60% by the fifth year after injury (representing a 40% decline in the proportion employed in the fifth year after injury compared with the year of injury). The similarity in the results of these studies with those of our study emphasizes the drastic economic consequences associated with traumatic injury and supports the generalizability of our results.

There are many explanations for the reduced reentry of SCI survivors into the labor force. Many SCI survivors do not return to the same level of preinjury functionality despite rehabilitation or additional medical therapy; consequently, they cannot perform the same tasks required in their preinjury employment. Patients may require assistive technology or mobility aids to be able to return to the workforce.^1,43,48,49^ Changes in employment status that survivors experience could be minimized through vocational rehabilitation services.^18,50,51^ The magnitude of employment loss and work earnings loss found in this national cohort should highlight the importance of vocational rehabilitation in this patient population.

### Strengths and Limitations

Our study has several key strengths. We used longitudinally linked health and personal income data sources that allowed us to evaluate long-term outcomes associated with SCI. We were therefore able to quantify the duration of income loss after injury, which is extremely important for guiding postinjury care pathways and the associated economic, health, and social policy to support individuals with disabilities after injury. Additionally, due to the single-payer structure of the Canadian health care system, most Canadian hospitalizations were captured in our database. Furthermore, our matched DID methodology coupled with our creation of the comparison group from the cohort of interest allowed us to adjust for unmeasurable confounders associated with the occurrence of an SCI and thereby offered estimates of the association of the traumatic event with various labor market sequelae.

This study also has some important limitations. First, we did not include individuals who did not file taxes. This exclusion probably had a minimal impact on our inferences given that non–tax filers in Canada ages 18 to 64 years comprise approximately 2% of this age group in Canada.^36^ In addition, any eligible patients who were injured in Quebec or the 3 Canadian territories were excluded due to a lack of data availability. These regions represent approximately 23% of the total Canadian population; importantly, there are no known programs or services in those locations to our knowledge that would significantly affect any outcomes in this study.^28^ Furthermore, administrative and tax data do not provide a holistic understanding of the disease state or type of employment. We do not have any specific granular data elements regarding disease specifications, such as information from a detailed neurological examination or data on underemployment, educational status, or type of occupation, that may help further delineate the association of SCI and labor market outcomes.^3^ Moreover, we analyzed only income derived from employment or self-employment earnings and did not include other potential sources of income, such as investment income or insurance payments. However, in the age group included in this study, individual earnings from employment represent a mean of 80% of the total income.^25^

## Conclusions

The loss of employment and income are important outcomes associated with traumatic cervical SCI for individuals with injuries and society. Costs to individuals and society represent a dimension of SCI that has been largely underreported in prior studies. At the individual level, tenuous employment and low income are associated with poor health outcomes. At the societal level, health-related disability is associated with the labor market through reduced productivity and increased absenteeism. In this study, we empirically modeled the association of SCI with individual income and employment status. We found that SCI was associated with changes in income and employment that were severe and persistent over the course of the 5 years after injury. Our findings add to the existing literature by quantifying the magnitude of labor market outcomes associated with cervical SCI. These findings provide important financial context to the care of patients with SCI that should inform policymakers and multidisciplinary acute and rehabilitation teams treating these patients.

## Notes

Supplement 1. **eMethods 1.** Datasets and Linkage **eTable 1.** Cervical SCI ICD-10-CA Diagnostic Codes **eFigure 1.** Cohort Creation Flowchart **eFigure 2.** Association Between Exposed and Comparison Cohorts **eMethods 2.** Matching **eFigure 3.** Event Study Results **eMethods 3.** Model Specification **eTable 2.** Summary of Individual and Injury Characteristics Stratified by Injury Status **eFigure 4.** Unmatched Cohort Income and Employment Over Time **eTable 3.** Difference-in-Difference in Individual Earnings and Employment for Unmatched Cohort
eReferences.

Supplement 2.
Data Sharing Statement

## References

[1] Al-Khodairy AT, el Masry WS. Vocational rehabilitation and spinal cord injuries. In: Gobelet C, Franchignoni F, eds. Vocational Rehabilitation: Collection de L’Académie Européenne de Médecine de Réadaptation. Springer; 2006. doi:10.1007/2-287-29745-6_11

[2] Pickett W, Simpson K, Walker J, Brison RJ. Traumatic spinal cord injury in Ontario, Canada. J Trauma. 2003;55(6):1070-1076. doi:10.1097/01.TA.0000034228.18541.D1 14676653

[3] Wilson JR, Cronin S, Fehlings MG, . Epidemiology and impact of spinal cord injury in the elderly: results of a fifteen-year population-based cohort study. J Neurotrauma. 2020;37(15):1740-1751. doi:10.1089/neu.2020.6985 32292120

[4] Pickett GE, Campos-Benitez M, Keller JL, Duggal N. Epidemiology of traumatic spinal cord injury in Canada. Spine (Phila Pa 1976). 2006;31(7):799-805. doi:10.1097/01.brs.0000207258.80129.03 16582854

[5] García-Altés A, Pérez K, Novoa A, . Spinal cord injury and traumatic brain injury: a cost-of-illness study. Neuroepidemiology. 2012;39(2):103-108. doi:10.1159/000338297 22846706

[6] Furlan JC, Fehlings MG. Cardiovascular complications after acute spinal cord injury: pathophysiology, diagnosis, and management. Neurosurg Focus. 2008;25(5):E13. doi:10.3171/FOC.2008.25.11.E13 18980473

[7] Sezer N, Akkuş S, Uğurlu FG. Chronic complications of spinal cord injury. World J Orthop. 2015;6(1):24-33. doi:10.5312/wjo.v6.i1.24 25621208 PMC4303787

[8] North NT. The psychological effects of spinal cord injury: a review. Spinal Cord. 1999;37(10):671-679. doi:10.1038/sj.sc.3100913 10557122

[9] Dale-Olsen H. Absenteeism, efficiency wages, and marginal taxes. Scand J Econ. 2013;115(4):1158-1185. doi:10.1111/sjoe.12028

[10] Mullen KJ, Rennane S. Worker absenteeism and employment outcomes: a literature review. National Bureau of Economic Research. Accessed May 17, 2024. https://www.nber.org/sites/default/files/2020-04/NB17-20%20Mullen,%20Rennane%20-%20QT2_0.pdf

[11] Howley P, Knight S. Staying down with the Joneses: differences in the psychological cost of unemployment across neighbourhoods. Work Employ Soc. 2022;36(6):1097-1117. doi:10.1177/09500170211003483

[12] Warr P. Psychological aspects of employment and unemployment. Psychol Med. 1982;12(1):7-11. doi:10.1017/S0033291700043221 7043521

[13] Priebe MM, Chiodo AE, Scelza WM, Kirshblum SC, Wuermser LA, Ho CH. Spinal cord injury medicine: 6. economic and societal issues in spinal cord injury. Arch Phys Med Rehabil. 2007;88(3)(suppl 1):S84-S88. doi:10.1016/j.apmr.2006.12.005 17321854

[14] Budd MA, Gater DR Jr, Channell I. Psychosocial consequences of spinal cord injury: a narrative review. J Pers Med. 2022;12(7):1178. doi:10.3390/jpm12071178 35887675 PMC9320050

[15] Janssen M, Heerkens Y, Kuijer W, van der Heijden B, Engels J. Effects of mindfulness-based stress reduction on employees’ mental health: a systematic review. PLoS One. 2018;13(1):e0191332. doi:10.1371/journal.pone.0191332 29364935 PMC5783379

[16] Modini M, Joyce S, Mykletun A, . The mental health benefits of employment: results of a systematic meta-review. Australas Psychiatry. 2016;24(4):331-336. doi:10.1177/1039856215618523 26773063

[17] Utzet M, Valero E, Mosquera I, Martin U. Employment precariousness and mental health, understanding a complex reality: a systematic review. Int J Occup Med Environ Health. 2020;33(5):569-598. doi:10.13075/ijomeh.1896.01553 32940256

[18] Borg SJ, Geraghty T, Arora M, . Employment outcomes following spinal cord injury: a population-based cross-sectional study in Australia. Spinal Cord. 2021;59(10):1120-1131. doi:10.1038/s41393-021-00639-z 34002015

[19] Leiulfsrud AS, Solheim EF, Reinhardt JD, . Gender, class, employment status and social mobility following spinal cord injury in Denmark, the Netherlands, Norway and Switzerland. Spinal Cord. 2020;58(2):224-231. doi:10.1038/s41393-019-0356-3 31575981

[20] Cao Y, Krause JS. Estimation of indirect costs based on employment and earnings changes after spinal cord injury: an observational study. Spinal Cord. 2020;58(8):908-913. doi:10.1038/s41393-020-0447-1 32139887

[21] Lidal IB, Huynh TK, Biering-Sørensen F. Return to work following spinal cord injury: a review. Disabil Rehabil. 2007;29(17):1341-1375. doi:10.1080/09638280701320839 17729082

[22] Statistics Canada. Social data linkage environment (SDLE): overview. Updated 2022-12-16. Accessed May 17. 2024. https://www.statcan.gc.ca/en/sdle/overview

[23] Canadian Institute for Health Information. Data quality documentation, discharge abstract database: current-year information, 2019-2020. Accessed May 17, 2024. https://www.cihi.ca/sites/default/files/document/dad-data-quality-current-year-information-2019-2020-en.pdf

[24] Statistics Canada. Technical reference guide for the annual income estimates for census families, individuals and seniors: T1 family file, final estimates, 2018. Accessed May 17, 2024. https://www150.statcan.gc.ca/n1/pub/72-212-x/72-212-x2020001-eng.htm

[25] Statistics Canada. Income of individuals by age group, sex and income source, Canada, provinces and selected census metropolitan areas. Accessed May 17, 2024. https://www150.statcan.gc.ca/t1/tbl1/en/tv.action?pid=1110023901

[26] Robson J, Schwartz S. Who doesn’t file a tax return: a portrait of non-filers. Can Public Policy. 2020;46(3):323-339. doi:10.3138/cpp.2019-063

[27] World Health Organization. International Statistical Classification of Diseases, Tenth Revision (ICD-10). 2nd ed. World Health Organization; 2004.

[28] Haas B, Jeon SH, Rotermann M, . Association of severe trauma with work and earnings in a national cohort in Canada. JAMA Surg. 2021;156(1):51-59. doi:10.1001/jamasurg.2020.4599 33112383 PMC7593875

[29] Haas B, Gomez D, Zagorski B, Stukel TA, Rubenfeld GD, Nathens AB. Survival of the fittest: the hidden cost of undertriage of major trauma. J Am Coll Surg. 2010;211(6):804-811. doi:10.1016/j.jamcollsurg.2010.08.014 21036070

[30] O’Hara NN, Slobogean GP, Klazinga NS, Kringos DS. Analysis of patient income in the 5 years following a fracture treated surgically. JAMA Netw Open. 2021;4(2):e2034898. doi:10.1001/jamanetworkopen.2020.34898 33555329 PMC7871192

[31] Halvorsen A, Steinsbekk A, Leiulfsrud AS, Post MWM, Biering-Sørensen F, Pape K. Labour market participation after spinal cord injury: a register-based cohort study. Spinal Cord. 2023;61(4):244-252. doi:10.1038/s41393-023-00876-4 36717734 PMC10070183

[32] Ramakrishnan K, Mazlan M, Julia PE, Abdul Latif L. Return to work after spinal cord injury: factors related to time to first job. Spinal Cord. 2011;49(8):924-927. doi:10.1038/sc.2011.16 21383761

[33] Krause JS, Terza JV, Saunders LL, Dismuke CE. Delayed entry into employment after spinal cord injury: factors related to time to first job. Spinal Cord. 2010;48(6):487-491. doi:10.1038/sc.2009.157 19935754

[34] Ripollone JE, Huybrechts KF, Rothman KJ, Ferguson RE, Franklin JM. Evaluating the utility of coarsened exact matching for pharmacoepidemiology using real and simulated claims data. Am J Epidemiol. 2020;189(6):613-622. doi:10.1093/aje/kwz268 31845719 PMC7368132

[35] Iacus SM, King G, Porro G. Causal inference without balance checking: coarsened exact matching. Polit Anal. 2012;20(1):1-24. doi:10.1093/pan/mpr013

[36] Sanmartin C, Reicker A, Dasylva A, . Data resource profile: the Canadian Hospitalization and Taxation database (C-HAT). Int J Epidemiol. 2018;47(3):687-687g. doi:10.1093/ije/dyy038 29590346

[37] Chetty R, Hendren N, Katz LF. The effects of exposure to better neighborhoods on children: new evidence from the moving to opportunity experiment. Am Econ Rev. 2016;106(4):855-902. doi:10.1257/aer.20150572 29546974

[38] Statistics Canada. Consumer Price Index (CPI) statistics, measures of core inflation and other related statistics—Bank of Canada definitions: table 18-10-0256-01. Accessed May 17, 2024. https://www150.statcan.gc.ca/t1/tbl1/en/tv.action?pid=1810025601

[39] Rambachan A, Roth J. An honest approach to parallel trends. Harvard University. Accessed May 17, 2024. https://scholar.harvard.edu/files/jroth/files/roth_jmp_honestparalleltrends_main.pdf

[40] Roth J. Pretest with caution: event-study estimates after testing for parallel trends. Am Econ Rev Insights. 2022;4(3):305-322. doi:10.1257/aeri.20210236

[41] Statistics Canada. Retirement age by class of worker, annual: table 14-10-0060-01. Accessed May 17, 2024. https://www150.statcan.gc.ca/t1/tbl1/en/tv.action?pid=1410006001

[42] R Core Team. A Language and Environment for Statistical Computing. R Foundation for Statistical Computing; 2021.

[43] Franceschini M, Pagliacci MC, Russo T, Felzani G, Aito S, Marini C; Italian Group for the Epidemiological Study of Spinal Cord Injuries. Occurrence and predictors of employment after traumatic spinal cord injury: the GISEM study. Spinal Cord. 2012;50(3):238-242. doi:10.1038/sc.2011.131 22124342

[44] Kader M, Perera NKP, Sohrab Hossain M, Islam R. Socio-demographic and injury-related factors contributing to activity limitations and participation restrictions in people with spinal cord injury in Bangladesh. Spinal Cord. 2018;56(3):239-246. doi:10.1038/s41393-017-0001-y 29093546

[45] Alizadeh A, Dyck SM, Karimi-Abdolrezaee S. Traumatic spinal cord injury: an overview of pathophysiology, models and acute injury mechanisms. Front Neurol. 2019;10:282. doi:10.3389/fneur.2019.00282 30967837 PMC6439316

[46] Conroy L, McKenna K. Vocational outcome following spinal cord injury. Spinal Cord. 1999;37(9):624-633. doi:10.1038/sj.sc.3100904 10490853

[47] Ottomanelli L, Lind L. Review of critical factors related to employment after spinal cord injury: implications for research and vocational services. J Spinal Cord Med. 2009;32(5):503-531. doi:10.1080/10790268.2009.11754553 20025147 PMC2792457

[48] Bezuidenhout L, Rhoda A, Moulaee Conradsson D, Theron F, Joseph C. Factors influencing employment among people with spinal cord injury in South Africa. Disabil Rehabil. 2023;45(26):4381-4387. doi:10.1080/09638288.2022.2151651 36447405

[49] Castle R. An investigation into the employment and occupation of patients with a spinal cord injury. Paraplegia. 1994;32(3):182-187. doi:10.1038/sc.1994.33 8008422

[50] Jongbloed L, Backman C, Forwell SJ, Carpenter C. Employment after spinal cord injury: the impact of government policies in Canada. Work. 2007;29(2):145-154. 17726290

[51] Marini I, Lee GK, Chan F, Chapin MH, Romero MG. Vocational rehabilitation service patterns related to successful competitive employment outcomes of persons with spinal cord injury. J Vocat Rehabil. 2008;28(1):1-13.

