# Supplementary material for: Traumatic Cervical Spinal Cord Injury and Income and Employment Status
Source: JAMA Netw Open. 2024 Jun 25;7(6):e2418468. doi: 10.1001/jamanetworkopen.2024.18468 (PMC11200142; doi:10.1001/jamanetworkopen.2024.18468)
Supplement: Supplement 1. — eMethods 1. Datasets and Linkage eTable 1. Cervical SCI ICD-10-CA Diagnostic Codes eFigure 1. Cohort Creation Flowchart eFigure 2. Association Between Exposed and Comparison Cohorts eMethods 2. Matching eFigure 3. Event Study Results eMethods 3. Model Specification eTable 2. Summary of Individual and Injury Characteristics Stratified by Injury Status eFigure 4. Unmatched Cohort Income and Employment Over Time eTable 3. Difference-in-Difference in Individual Earnings and Employment for Unmatched Cohort eReferences. [file jamanetwopen-e2418468-s001.pdf]

Supplemental Online Content

Jaffe RH, Coyte PC, Chan BC-F, et al. Traumatic cervical spinal cord injury and income and employment status. *JAMA Netw Open*. 2024;7(6):e2418468. doi:10.1001/jamanetworkopen.2024.18468

eMethods 1. Datasets and Linkage

eTable 1. Cervical SCI ICD-10-CA Diagnostic Codes

eFigure 1. Cohort Creation Flowchart

eFigure 2. Association Between Exposed and Comparison Cohorts

eMethods 2. Matching

eFigure 3. Event Study Results

eMethods 3. Model Specification

eTable 2. Summary of Individual and Injury Characteristics Stratified by Injury Status

eFigure 4. Unmatched Cohort Income and Employment Over Time

eTable 3. Difference-in-Difference in Individual Earnings and Employment for Unmatched Cohort

eReferences.

This supplemental material has been provided by the authors to give readers additional information about their work.

© 2024 Jaffe RH et al. *JAMA Network Open.*

eAppendix 1. Methods: Datasets and Linkage

Data Overview

Discharge Abstract Database

Hospital records were be derived from the Canadian Institute for Health Information (CIHI) Discharge Abstract Database (DAD), which captures all acute care hospitalizations in the country except those in the province of Quebec for the fiscal years of 2004/2005 to 2019/2020.[1] Individuals were identified within each hospital admission record by their Health Insurance Number (HIN). This number is unique within provinces and territories. CIHI validates the dataset yearly as well as obtains the data directly from clinical chart abstractions manually performed in every Canadian hospital using standardized data definitions.

The DAD collects demographic, administrative, and clinical data for all hospitalizations at the individual level. To subset a cohort of SCI patients, we used International Classification of Diseases, Tenth Revision (ICD-10) diagnostic codes.[2]

T1-Family File

Longitudinal individual tax information was derived from the T1 Family File (T1FF) provided by the Canada Revenue Agency.[3] These data cover all persons who completed a T1 tax return or who received Federal child benefits, their non-filing spouses, their non-filing children identified from three sources (a file pertaining to Federal Child Benefits, the births files, and an historical file) and filing children who reported the same address as their parent.[3] All Canadians must file a tax return if they have any taxable income, capital gains, self-employment, or payments from or into any type of retirement account.[4] However, approximately 75% of all Canadians file tax returns every year and this rate varies by age. 98% of people over the age of 19 file tax returns compared to 64% of those aged exactly 18.[4]

Individuals are identified within the TIFF by social insurance numbers (SINs). The database is comprised of all records for years 2004 to 2019, where the year begins on January 1st and ends on December 31st.

Linkage

Statistics Canada has a repository of individual identifiers for all administrative data stored in the SDLE Key Registry that is linked to the DRD. The keys found in the SDLE key registry include CIHI-DAD record identifiers and the Social Insurance Numbers of CIHI-DAD individuals found in the DRD. The Social Insurance Numbers are used to link individual T1FF records to DAD information.

© 2024 Jaffe RH et al. *JAMA Network Open.*

eTable 1. Cervical SCI ICD-10-CA Diagnostic Codes

ICD-10-CA	Description
S14.10	Complete lesions of the cervical spinal cord
S14.1X	Incomplete lesions of the cervical spinal cord
S14.11	Central Cord Lesion of cervical spinal cord
S14.12	Anterior cord syndrome of cervical spinal cord
S14.13	Posterior cord syndrome of cervical spinal cord
S14.18	Other injuries of cervical spinal cord
S14.19	Unspecified injuries of cervical spinal cord

© 2024 Jaffe RH et al. *JAMA Network Open.*

eFigure 1. Cohort Creation Flowchart

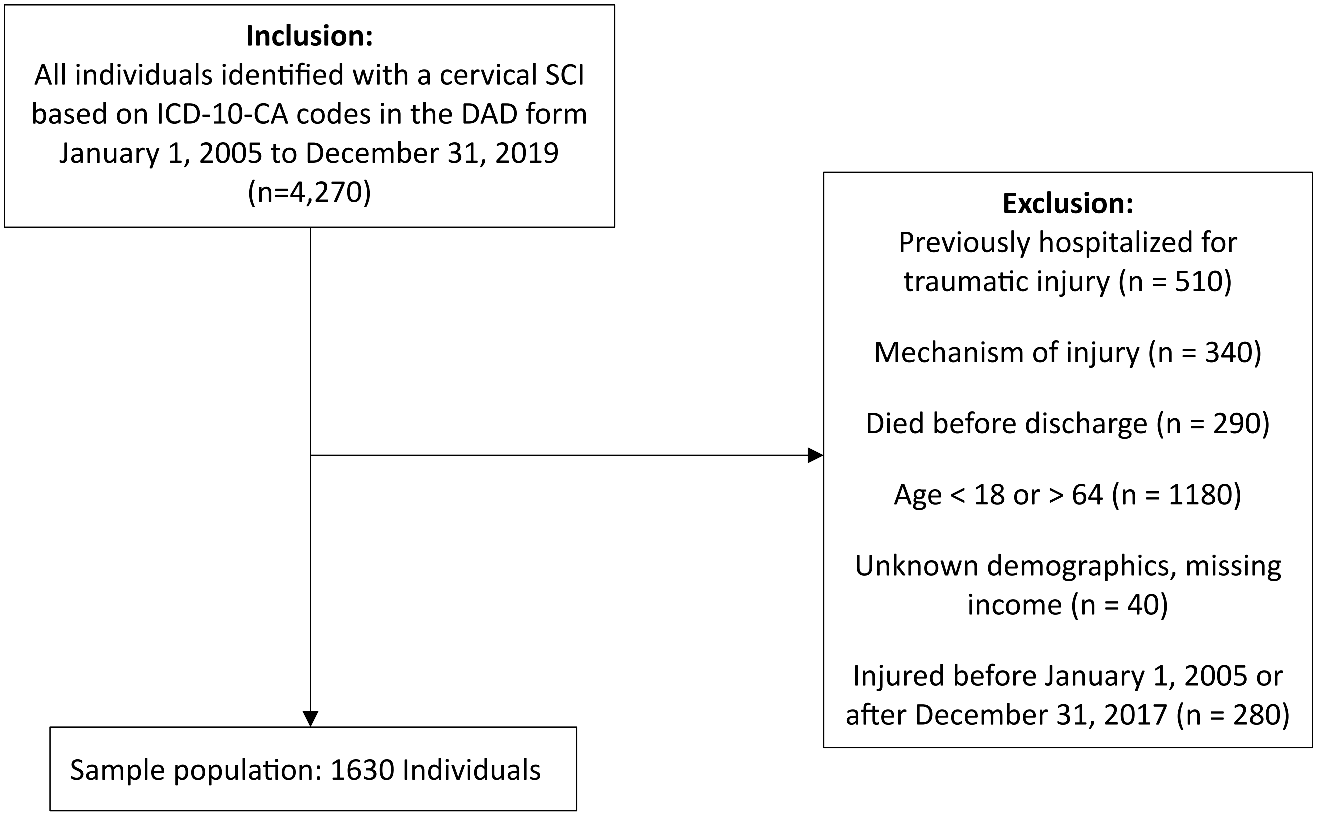

Flowchart illustrating the process of cohort creation, including exclusion criteria to generate the final cohort for analysis.

© 2024 Jaffe RH et al. *JAMA Network Open.*

eFigure 2. Relationship between Exposed and Comparison Cohorts

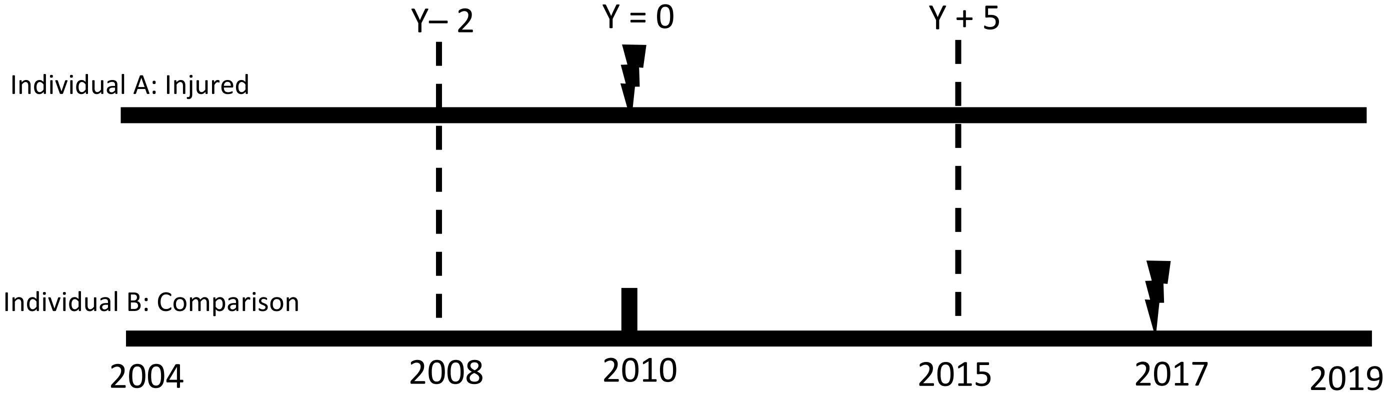

The comparison group was created from the main injury cohort. The window of observation for individual A, who was injured in 2010, is from 2008 (Y-2) to 2015 (Y+5). Individual B is a comparison individual because their injury occurred at least six years after Individual A's injury. Individual B tax information between 2008 and 2015 will be compared to Individual A's data.

© 2024 Jaffe RH et al. *JAMA Network Open.*

eAppendix 2. Methods: Matching

CEM Design

We used Coarsened Exact Matching (CEM) because of its advantage of reducing bias into treatment effect within large datasets with many covariates.[5] With CEM, the user will stratify their data into pre-specified 'bins' of covariates to match on. Observations within the bins will be matched based on these categories.

The number of bins we used were based on the Sturges' Rule that determines an optimal bin number relative to the size of the dataset.[6] It follows the formula:

$$Optimal\ Bins = [\log_2 n + 1]$$

Where n represents the number of observations. The optimal bins for our dataset to match on was 11 bins for each covariate.

© 2024 Jaffe RH et al. *JAMA Network Open.*

eFigure 3. Event Study Results

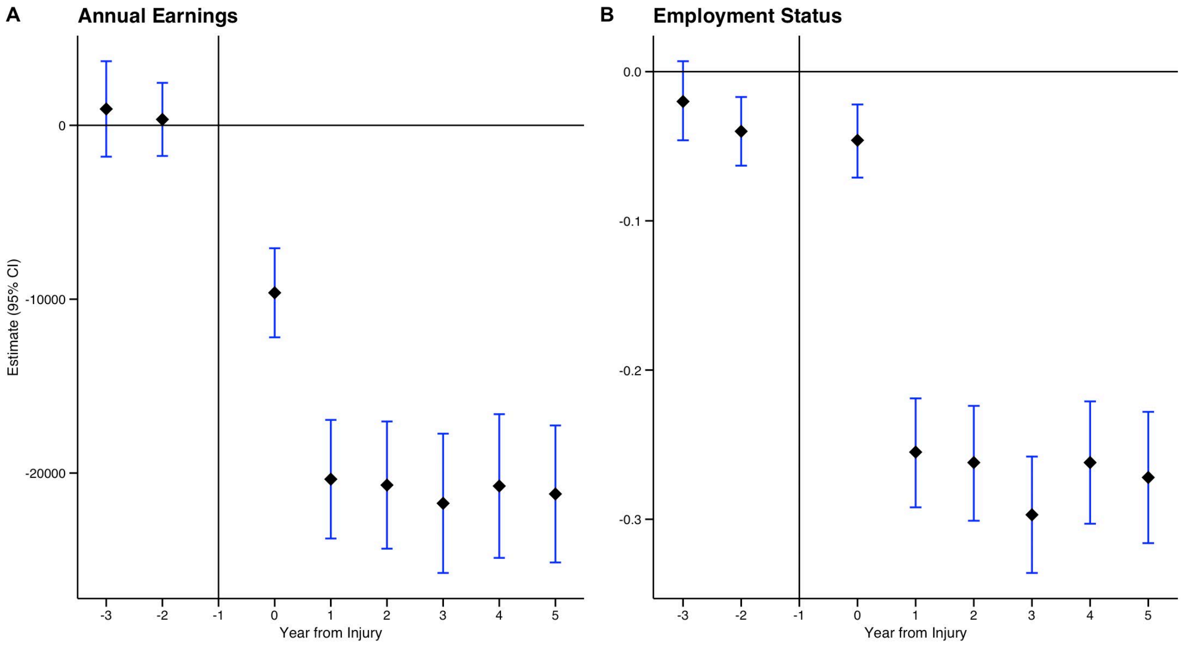

The figure shows the results of an event study model that aims to support the parallel trends assumption for difference-in-difference analyses.

© 2024 Jaffe RH et al. *JAMA Network Open.*

eAppendix 2. Methods: Model Specification

Model Specification

We use two generalized mixed models to control for individual level effects over time. The first model is a linear mixed model to estimate the annual income change:

$$Y_{ij} = \beta_{0ij} + \beta_1 Injured_i + \beta_2 Time_j + \beta_3 (Injured * Time)_{ij} + \beta_4 Age_{ij} + \beta_5 FiscalYear_{ij} + \epsilon_{ij}$$

Where i is the individual. j represents the time before (0) or after injury (1), where time before is always the year prior to injury. Age is a continuous variable, whereas fiscal year is a set of dummy variables.

The second model to estimate the change in employment status is a generalized mixed model with a probit link. It is the same as the linear mixed model, however, since employment status takes on a binary status, the outcome is now modelled by a probit distribution.

$$Y_{ij} = \beta_{0ij} + \beta_1 Injured_i + \beta_2 Time_j + \beta_3 (Injured * Time)_{ij} + \beta_4 Age_{ij} + \beta_5 FiscalYear_{ij} + \epsilon_{ij}$$

$$s.t. \quad E\left(Y_{ij}\right) \sim \phi^{-1}(\pi_{ij})$$

© 2024 Jaffe RH et al. *JAMA Network Open.*

eTable 2. Summary of Individual and Injury Characteristics Stratified by Exposure

	Unmatched		Matched (Weighted)		
	Patients, No. (%)				
**Variable**	**Exposed (n = 1630)**	**Comparison (n = 1100)[a]**	**Exposed (n = 790)**	**Comparison (n = 620)[a]**	**Standardized Diff.**
**Individual Characteristics**					
Age, mean (SD)	47 (12.62)	42 (10.54)	43 (10.38)	43 (11.96)	0.02
Urban Residency	1255 (77)	869 (79)	624 (79)	508 (82)	0.09
Male	1304 (80)	869 (79)	687 (87)	539 (87)	0
Income (Y = -2), mean (SD)	47000 (73866.02)	51000 (81885.50)	46000 (48251.50)	45000 (43174.22)	0.02
Income (Y = -1), mean (SD)	48000 (91816.90)	52000 (87646.00)	46000 (49262.22)	46000 (43554.98)	0
Family Size, mean (SD)	2.50 (1.41)	2.80 (1.46)	2.60 (1.43)	2.80 (1.42)	0.15
Married	783 (48)	583 (53)	403 (51)	316 (51)	0
Self Employed	196 (12)	143 (13)	55 (7)	43 (7)	0
**Injury Characteristics[b]**					
Discharge Disposition					
Transferred to a long-term care facility	650 (40)	242 (22)	332 (42)	130 (21)	
Discharged to a home setting	603 (37)	407 (37)	292 (37)	223 (36)	
Transferred to another facility providing inpatient care	293 (18)	374 (34)	126 (16)	210 (34)	
Discharged to a home setting with support services	49 (3)	55 (5)	40 (5)	56 (9)	
Transferred to other, or signed out (against medical advice)	33 (2)	22 (2)	419 (53)	322 (52)	
Province of Injury					
Ontario	668 (41)	462 (42)	198 (25)	155 (25)	
British Columbia	424 (26)	275 (25)	95 (12)	74 (12)	
Alberta	212 (13)	132 (12)	40 (5)	56 (9)	
PEI, NL, NS, NB, Manitoba[c]	212 (13)	154 (14)	47 (6)	192 (5)	

© 2024 Jaffe RH et al. *JAMA Network Open.*

Length of Stay (Days), mean (SD)	27 (74.55)	23 (72.12)	25 (67.48)	22 (69.20)	
Responsibility for Payment					
Prov/Terr. Responsibility	1467 (90)	1011 (91)	719 (91)	570 (92)	
WCB/WSIB, Other Prov/Terr (resident of Canada), Canadian Resident Self-pay, or other	82 (5)	77 (7)	71 (9)	50 (8)	
Mechanism of Injury					
Fall	848 (52)	605 (55)	419 (53)	353 (57)	
Motor vehicle traffic	293 (18)	165 (15)	142 (18)	99 (16)	
Struck by object, or cut/pierce	163 (10)	77 (7)	71 (9)	37 (6)	
Other transportation	114 (7)	99 (9)	63 (8)	50 (8)	
Pedal cyclist (incl. pedestrian)	98 (6)	66 (6)	40 (5)	43 (7)	
Natural/environmental, Machinery, Firearm, or other	130 (8)	77 (7)	55 (7)	43 (7)	
Complete Injury [d]	212 (13)	88 (8)	103 (13)	50 (8)	

[a]Refers to the number of unique individuals represented in the comparison group. Prior to matching there were 21,155 person-years of patient income for the exposed group and 46,573 person-years for the comparison group. After matching, 10,933 person-years were compared to 31,090 person-years for the comparison group.
[b]Characteristics of the future injury for the comparison group patient. [c]NL = Newfoundland; PEI = Prince Edward Island; NS = Nova Scotia; NB = New Brunswick, Territories = Northwest Territories, Yukon, Nunavut
[d]Complete injury refers to an ASIA A injury.

Note: all variables assessed at corresponding exposure year (or index year if exposed group)

eFigure 4. Unmatched Cohort Income and Employment Overtime

© 2024 Jaffe RH et al. *JAMA Network Open.*

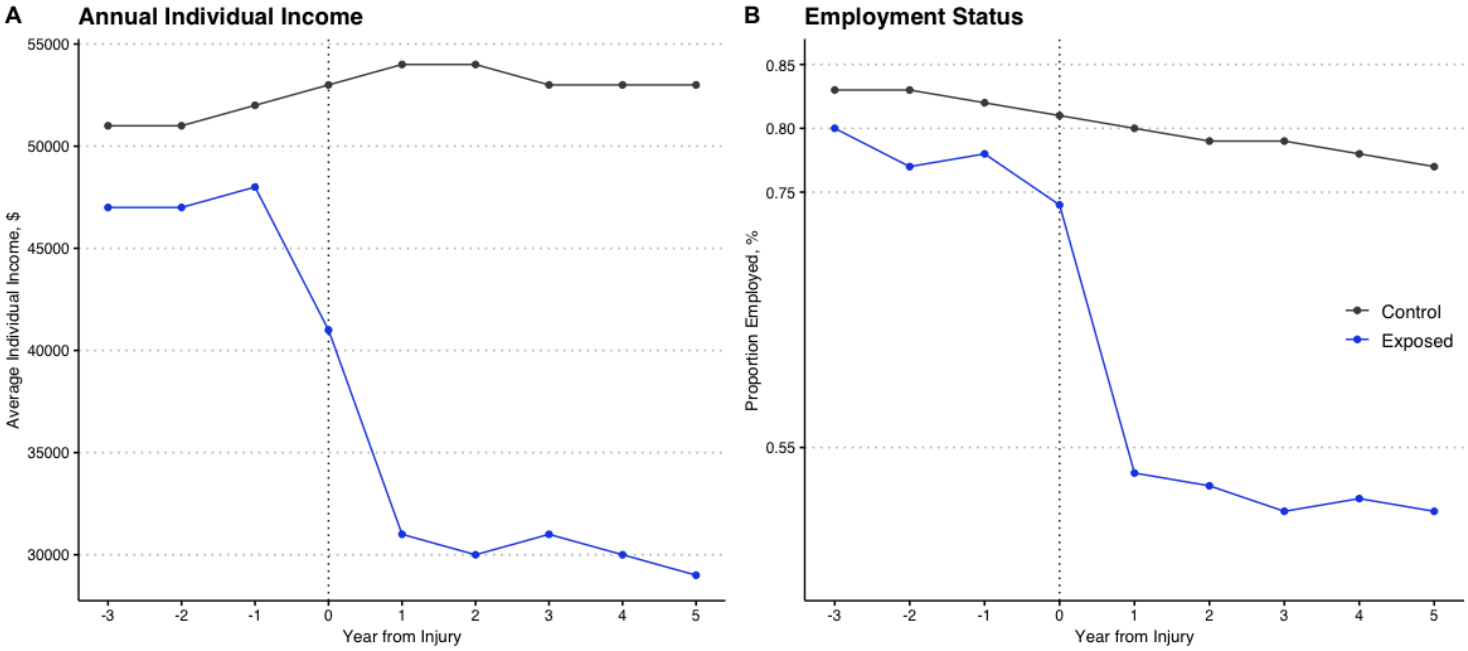

The figure shows the unweighted average annual income (A) and the proportion employed (B) for the entire SCI cohort from 3 years prior to injury until the 5th year post injury.

© 2024 Jaffe RH et al. *JAMA Network Open.*

eTable 3. Difference-in-Difference in Individual Earnings and Employment for Unmatched Cohort

Cohort	Year From Injury	Exposure Group	Unexposed Group	Difference-in-Difference (95% CI)[a]
**Annual Income (Mean (SD), $ CAD)**				
Unmatched	-1	48000 (91693)	52000 (87556)	NA
	1	31000 (79033)	54000 (99069)	-20286 (-24149, -16422)
	2	30000 (83850)	54000 (106377)	-20574 (-24554, -16594)
	3	31000 (81123)	53000 (104667)	-21787 (-26615, -16960)
	4	30000 (71041)	53000 (100014)	-21752 (-26951, -16553)
	5	29000 (67287)	53000 (99363)	-22776 (-27874, -17677)
**Proportion Employed**				
Unmatched	-1	78	82	NA
	1	53	80	-18.8 (-20, -16)
	2	52	79	-19.9 (-21, -17)
	3	50	79	-19.3 (-22, -18)
	4	51	78	-19.5 (-22, -17)
	5	50	77	-18 (-22, -17)

[a]Difference-in-difference estimates from the year prior to injury to the year indicated for patients with injuries and comparison participants, derived from multivariable modelling.

© 2024 Jaffe RH et al. *JAMA Network Open.*

eReferences. Supplemental References

1. CIHI. Data Quality Documentation, Discharge Abstract Database — Current-Year Information, 2019-2020. 2020.
2. World Health Organization. International statistical classification of diseases and related health problems. 10th revision, 2nd edition. ed. Geneva: World Health Organization; 2004.
3. Canada S. Technical Reference Guide for the Annual Income Estimates for Census Families, Individuals and Seniors T1 Family File, Final Estimates, 2018. 2018.
4. Sanmartin C, Reicker A, Dasylva A, Rotermann M, Jeon SH, Fransoo R, et al. Data Resource Profile: The Canadian Hospitalization and Taxation Database (C-HAT). Int J Epidemiol. 2018;47(3):687-g.
5. Iacus S, King G, Porro G. Causal inference without balance checking: Coarsened exact. Political Analysis. 2012;20(1).
6. Sturges HA. The Choice of a Class Interval. Journal of the American Statistical Association. 1926;21(153):65-6.

© 2024 Jaffe RH et al. *JAMA Network Open.*
