# Supplementary material for: Traumatic Cervical Spinal Cord Injury and Income and Employment Status
Source: JAMA Netw Open. 2024 Jun 25;7(6):e2418468. doi: 10.1001/jamanetworkopen.2024.18468 (PMC11200142; doi:10.1001/jamanetworkopen.2024.18468)
Supplement: Supplement 2. — Data Sharing Statement [file jamanetwopen-e2418468-s002.pdf]

Data Sharing Statement

Jaffe. Traumatic Cervical Spinal Cord Injury and Income and Employment Status. *JAMA Netw Open*. Published online June 25, 2024. doi:10.1001/jamanetworkopen.2024.18468

Data
Data available: No
